# Development of a multi-level family planning intervention for couples in rural Uganda: key findings & adaptations made from community engaged research methods

**DOI:** 10.1186/s12905-023-02667-8

**Published:** 2023-10-21

**Authors:** Christine Muhumuza, Katelyn M. Sileo, Rhoda K. Wanyenze, Trace S. Kershaw, Haruna Lule, Samuel Sekamatte, Susan M. Kiene

**Affiliations:** 1 Department of Epidemiology and Biostatistics, School of Public Health, Makerere University, Kampala, Uganda; 2 Department of Public Health, University of Texas at San Antonio, San Antonio, TX USA; 3 Department of Disease Control and Environmental Health, School of Public Health, Makerere University, Kampala, Uganda; 4 Department of Social and Behavioral Sciences, Yale School of Public Health, New Haven, CT USA; 5 Gombe Hospital, Butambala Local Government, Kayenje, Uganda; 6 School of Public Health, San Diego State University, San Diego, USA

**Keywords:** Contraception, Family Planning, Reproductive Health, Intervention, Uganda

## Abstract

**Background:**

Uganda has among the highest fertility rates in the world and multi-level barriers contribute to the low contraceptive use.

**Objective:**

The objective of this study was to develop a culturally and socially relevant, community-based intervention to increase contraceptive use among couples in rural Uganda through community-engaged research methods. This study reports on the community-engaged research that informed the intervention’s content and structure and the final content of the intervention; the evaluation of the pilot intervention will be reported upon completion.

**Methods:**

An intervention steering committee of community stakeholders reviewed the initially proposed intervention content and approach. Four (4) gender-segregated focus groups were conducted with twenty-six (26) men and women who had an unmet need for family planning. Fifteen key-informant interviews were conducted with community leaders and family planning stakeholders. Finally, the 4-session intervention was pilot tested with a cohort of couples (N = 7) similar in demographics to the target sample of the future pilot intervention trial. Qualitative data were analyzed thematically.

**Results:**

Findings included the identification of community beliefs to reshape in order to increase family planning acceptance, as well as strategies to engage men, acceptable approaches for community leader involvement in the intervention to endorse family planning, and methods for managing gender dynamics and minimizing risk of unintended negative consequences of participation. The findings were used to inform the ideal structure and format of the intervention, including the distribution of contraceptives directly during group sessions, and identified the need to strengthen health worker capacity to provide Long-Acting Reversable Contraceptives (LARCs) as part of the intervention.

**Conclusions:**

These findings were used to refine an intervention before a larger scale pilot test of its feasibility, acceptability, and potential efficacy. They can inform other multi-level family planning interventions in similar settings and the methods can be adopted by others to increase the feasibility, acceptability, and cultural relevance of interventions.

## Introduction

It is well-established that women face significant barriers to contraceptive use in low and middle-income countries (LMICs), including those at the individual, interpersonal, and community-levels, such as individual knowledge deficits and fear of side-effects, [1–4] male partner, peer, and family influence, [5–7] and social cultural norms that promote large family size and traditional gender roles [8–11]. At the health-system-level, numerous other structural barriers can simultaneously impede family planning service access, such as long wait times, limited contraceptive mix, stock-outs, lack of provider training in long-acting reversable methods (LARCs), and geographic distance and transportation barriers, especially in rural areas [12]. Accordingly, there have been numerous calls among researchers and family planning programmers for the development and implementation of multilevel interventions to address family planning needs, however, few interventions have incorporated a multilevel intervention approach to-date [13].

In this manuscript, we describe the development of a multi-level, community-based intervention aimed to increase contraceptive use among couples with an unmet need for family planning in rural Uganda. In 2021, Uganda had the seventh highest fertility rate in the world at 5.45 children per woman, [14] and 30.5% of married women had an unmet need for modern contraceptives in 2020 [15]. Unmet need refers to the gap between women’s reproductive intentions and their contraceptive behaviour (i.e., wanting to delay pregnancy but not using effective methods to do so). Similar to those previously described for LMICs, Ugandan women are faced with multilevel barriers to contraceptive use that span the individual to societal level, which were highlighted in the preliminary research that informed our intervention [16–18]. This research was conducted using both qualitative and quantitative methods with women and men from the same rural area of central Uganda as the planned intervention. It corroborated the need for a multilevel approach to family planning promotion by highlighting misinformation, partner and community approval, relationship dynamics, cultural norms, as well as health-system barriers as family planning determinants [16–18]. This research also highlighted the need to engage men by bringing services to the community, [17] and identified gender-specific family planning facilitators: financial benefits and child health were motivators for men, [17] while the health benefits of child spacing and desire to increase relationship equity through couples counseling were motivators for women [17].

Based on these data, the investigative team conceptualized the *Family Health = Family Wealth* intervention, a multi-level intervention aimed to engage *both* men and women by promoting family planning’s benefits to “family wealth” (physical, relationship, economic well-being), while highlighting the need to reshape community norms that dictate family size preference. Based on the need for a multilevel approach, and a particular need for normative change around gender inequitable norms that influence large family size preference and gender dynamics that prohibit women’s autonomous use of contraceptives, the investigative team conceptualized the intervention as four facilitated group sessions with couples (two gender separate, two gender mixed) that would integrate a community dialogue approach to reshape social norms. The community dialogue’s effect would be enhanced by integrating multilevel content to improve knowledge, motivation, self-efficacy, relationship dynamics, and health-system barriers, tailored to the needs of men and women.

Community dialogues follow a defined process to identify local drivers of sexual and reproductive health with community groups, [19] and engage the community in problem-solving towards a common issue through community-based participatory methodologies [20]. This approach is commonly grounded in Campbell and Cornish’s social psychological theory of transformative communication, [21] which emphasizes the role of conversations in safe social spaces in the development of social norms [22]. The dialogue that takes place allows community members to critically think about social norms underpinning a community problem, [23] and reconstruct community norms together, creating social environments that promote healthy behavior [24].

**Fig. 1:**
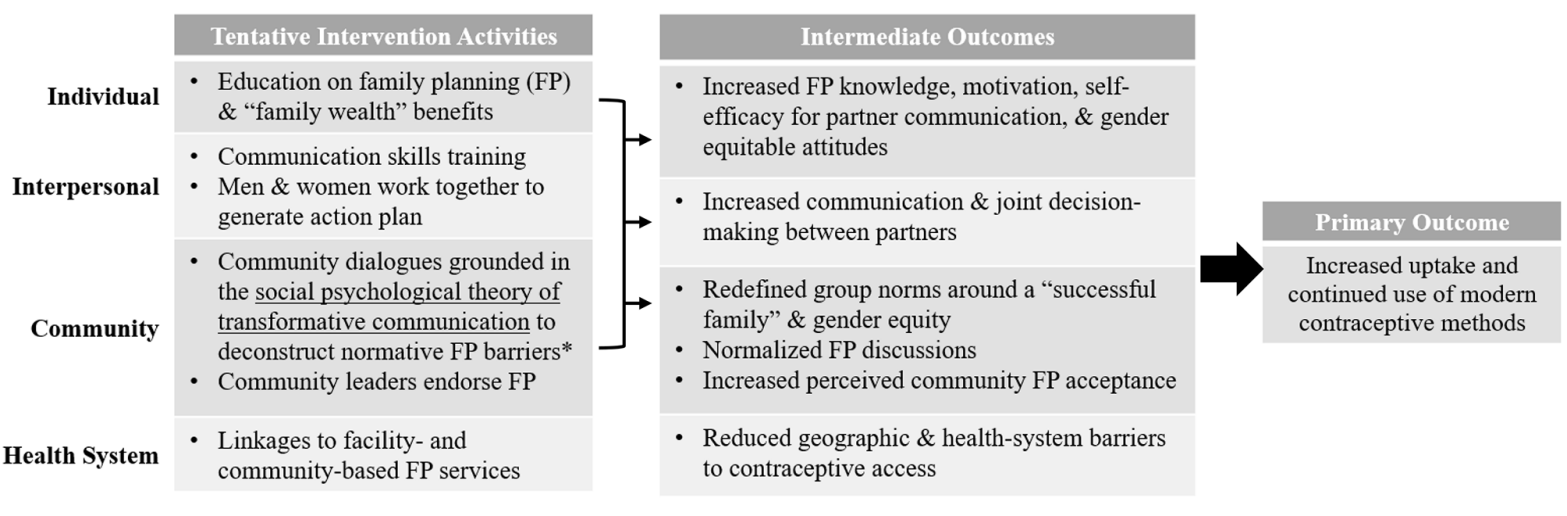
Conceptual Model of the “Family Health = Family Wealth” Intervention’s Effect of Contraceptive Use by Level of the Social Ecological Model with Tentative Intervention Activities Note: The primary mechanism of action theorized to affect change across the individual, interpersonal, and community-levels is community dialogues grounded in Campbell and Cornish’s social psychological theory of transformative communication. Other content across ecological levels is tentatively included to address other multilevel barriers to contraceptive use. Content is subject to change based on the findings of community-engaged research methods to elicit community feedback on the intervention content’s feasibility, acceptability, and potential to influence locally relevant barriers to contraceptive use

Based on our preliminary research, we aimed to include community dialogues grounded in the social psychological theory of transformative communication [21] to reshape gender inequitable norms and the definition of a “successful family.” In the intervention’s conceptual model, transformative communication is positioned as the primary mechanism of action to affect change across social ecological levels, specifically through change in individual attitudes, interpersonal communication, the perception of community norms related to family planning acceptance and gender equity, and reduced health-system barriers to contraceptive use. See Fig. 1 for a depiction of the original conceptual model for the intervention’s effect on contraceptive use, integrating the social psychological theory of transformative communication with the social ecological model that together guide the intervention.

The initially proposed content beyond transformative communication aimed to address knowledge, motivation, self-efficacy, relationship dynamics, and health-system barriers are also highlighted in the Fig. 1. In summary, they included: family planning education delivered by a local health worker, relationship-building through communication skills training, shared decision-making activities, modeling of gender equitable couples, economic skills training (to engage men’s interest, while increasing equity and shared decision-making within the couple) delivered by the study intervention facilitators, family planning and program endorsement by local leaders, and the development of “Family Action Plans” and “Community Action Plans” delivered by the study intervention facilitators. It is common in community dialogues for the group to work together to develop a “Community Action Plan” to elicit community-derived solutions to the problem of focus that utilize existing resources, and increase community ownership of these solutions [25]. We planned to engage participants in creating family planning-focused Community Action Plans, and adapt this concept into Family Action Plans for couples to work on their own health, relationship, and economic goals. Finally, we planned to create linkages between the health system and community to reduce structural barriers to contraceptive use by integrating local health workers into the program itself (midwives, village health teams [VHTs]), and planned to explore the acceptability and feasibility of the direct distribution of contraceptive methods during the group sessions.

After the initial conceptualization of *Family Health = Family Wealth*, we conducted a series of community-engaged inquiries to further develop the intervention’s content and structure, eliciting feedback on how to tailor it to the needs of the local population and community/health-system setting. In this manuscript, we report the findings of these community engaged methods and how they informed the resulting intervention package that was implemented and evaluated in the intervention’s pilot trial (recently reported elsewhere) [26].

## Methods

The study was conducted in selected rural and peri-urban communities of Butambala District, central Uganda located approximately two hours from the capital city of Kampala. The investigative team had been engaging in collaborative research in this area for more than 10 years. Family planning services in this district are integrated into general outpatient services and are provided for free in all public health facilities. Family planning services are also provided by private not-for-profits (PNFPs) and faith-based PNFP facilities, which mainly promote natural methods (i.e., counting days). The public health facilities follow Uganda’s five level decentralized health system structure (I-IV). Health Center IIs and above offer condoms, oral pills, and injectable contraceptives. Health Centre IIIs and above offer intrauterine devices (IUDs) and implants, and Health Center IVs provides non-reversible methods (vasectomy, tubal ligation). Village Health Teams (VHTs), a cadre of community health workers, serve as liaisons between the community and health facilities, and support community family planning efforts. VHTs provide community education about family planning and distribute short-term methods (condoms, oral pills) directly in the community. Also, an international nongovernmental organization, Marie Stopes, provides regular community outreach for all contraceptive methods in selected villages within the district. The villages in this district are mostly homogenous in demographics and size with only small commercial centers (no city within the district).

### Community-engaged methods for intervention refinement

**Fig. 2:**
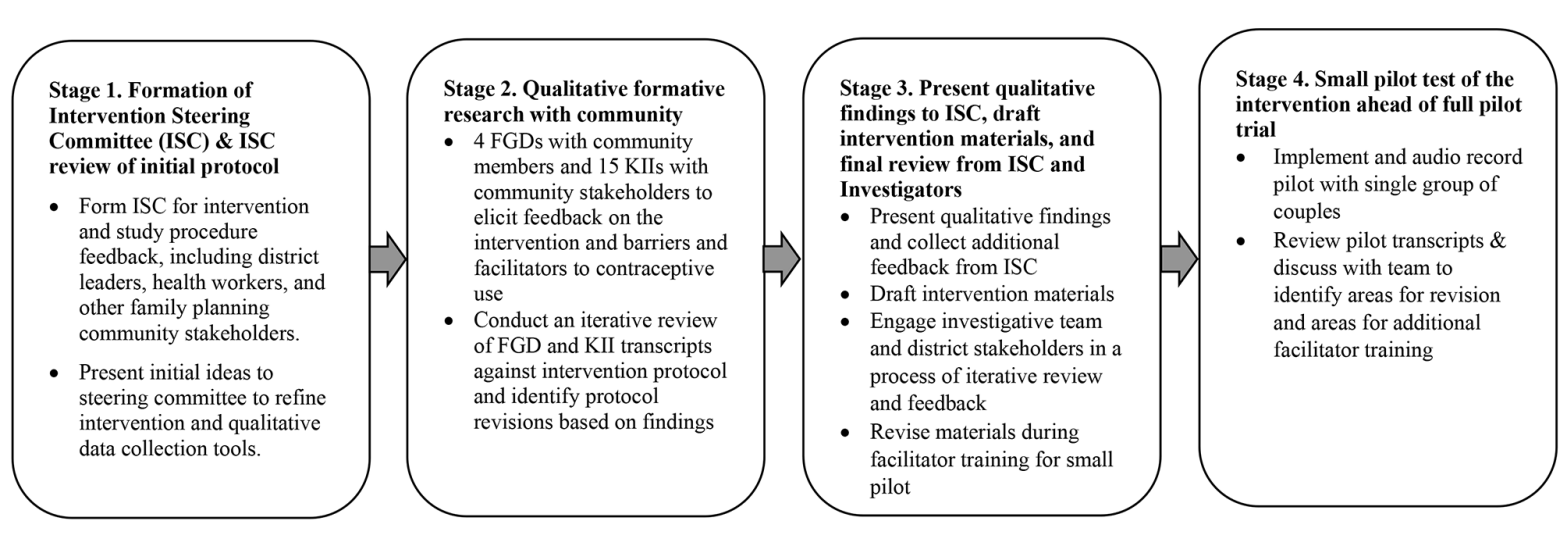
Chronological Overview of Community-Engaged Methods used to for Intervention Development Notes: ISC = Intervention Steering Committee; FGDs = Focus group discussions; KIIs = Key informant interviews

A visual depiction of the community-engaged research methods used to gather feedback on and further develop the *Family Health = Family Wealth* intervention is presented in Fig. 2 to illustrate the overall timeline of study procedures, described in detail below. All study procedures were reviewed and approved by the Institutional Review Boards (IRBs) at the University of Texas at San Antonio (protocol # 19–253, October 2019) and Makerere University School of Public Health (protocol # 748, January 2020). The study was also approved by the Uganda National Council for Science and Technology (May 2020) and by Butambala District Health leadership, who provided formal project endorsement, entry into the health centers in the district, and introductions to key stakeholders for qualitative data collection. Subsequently, two qualitative interviewers familiar with the area of study, the Luganda local language, and experienced in qualitative research methods were hired and trained to assist in the data collection process.

Stage 1 of the intervention development process began with assembling an intervention steering committee (ISC) tasked to guide the tailoring of the intervention to the local community and health system context and to linking the study team to the local communities, clinics, and other stakeholders essential to study progress. The ISC was made up of district health officials, family planning providers, VHTs, and other community stakeholders. Ahead of the planned qualitative data collection, the investigative team first presented the proposed intervention protocol and research plan to the ISC in an in-person meeting in March 2020 to gather initial feedback and begin early refinement of the intervention. This meeting helped to raise issues that needed to be explored further in the planned formative research phase with the community participants (Stage 2 in Fig. 2, described next), and thus informed our interview and focus group tools.

Following a three-month government-mandated COVID-19 lockdown that temporarily halted all research activities (March-June 2020), the formative phase of the research began in June 2020 with the aim of drawing feedback on the intervention content and study procedures from relevant community stakeholders and community members. The research team developed and refined all qualitative data collection tools, which included questions on overall barriers and facilitators to contraceptive use in the local setting (relevant for developing intervention content), as well as questions to elicit feedback on the feasibility and acceptability of the planned intervention approach. In consultation with the ISC, we identified communities for our formative data collection as part of the process of selecting communities for the future intervention trial, aiming to identify communities that were similar across key characteristics. The communities identified were matched on population size (~ 2000), distance to health facilities offering contraceptives, and other contextual factors (e.g., demographics, distance to a trading center).

Four approximately one-hour focus group discussions (FGDs) were conducted with 26 community members (13 women, 13 men), stratified by age and gender (men < 30, men < 30, women < 30, women > 30). Focus groups were moderated by a trained facilitator experienced in qualitative research and a researcher assistant took detailed notes, used later to aid transcription.

Inclusion criteria included being from the selected communities, being of reproductive age (women: 18–40, or an emancipated minor, defined as individuals below 18 years who are married, have a child, or are self-sufficient; men: 18–50 or an emancipated minor), considering oneself married, speaks Luganda, not currently pregnant, and having an unmet need for modern contraceptive methods. An unmet need for family planning was defined as wanting to delay pregnancy for at least a year but not currently using a high-efficacy method of modern contraception; methods (tailored to availability in the local setting) included oral pills, intrauterine device, implants, injectables, and condom use 100% of the time. Since contraceptive uptake among those with an unmet is the goal of the intervention, those already using non-reversible methods (tubal ligation, vasectomy) were not included, as their need is met. While natural methods of contraception (e.g., counting days) can be high-efficacy when used correctly and can be an appropriate person-centered outcome, we only considered high-efficacy methods given that many women use natural methods because of low knowledge and access barriers to modern methods in the local area. FGD participants were compensated 22,000 Ugandan Shillings (~ 6 USD) for their time. See Table 1 for an overview of focus group participant characteristics. The final sample’s demographics (largely Muganda tribe, Muslim as well as Catholic and Protestant religion, and low lifetime experience with modern contraceptives) is representative of the communities selected for the intervention.

**Table 1:** Participant characteristics, focus group discussion participants, Uganda, 2020

	Total (N = 26) n (%) / Mean (SD)	Women (n = 13) n (%) / Mean (SD)	Men (n = 13) n (%) / Mean (SD)
Age	32.27 (10.4)	32.54 (9.8)	32.00 (11.3)
Tribe			
Muganda	24 (92.3%)	13 (100.0%)	11 (84.6%)
Munyarwanda	2 (7.7%)	0 (0.00%)	2 (15.4%)
Religion			
Muslim	18 (69.2%)	10 (76.9%)	8 (61.5%)
Catholic	4 (15.4%)	1 (7.7%)	3 (23.1%)
Protestant	4 (15.4%)	2 (15.4%)	2 (15.4%)
Education			
No grade	4 (15.4%)	2 (15.4%)	2 (15.4%)
Primary	15 (57.7%)	7 (53.8%)	8 (61.5%)
Secondary	6 (23.1%)	3 (23.1%)	3 (23.1%)
Tertiary	1 (3.8%)	1 (7.7%)	0 (0.0%)
Years married	13.46 (10.1)	14.31 (9.78)	12.62 (10.8)
Number of living children	5.73 (4.1)	5.15 (3.7)	6.31 (4.5)
Number of wives			
1	10 (38.5%)	4 (30.8%)	6 (46.2%)
2	13 (50.0%)	7 (53.8%)	6 (46.2%)
3	3 (11.5%)	2 (15.4%)	1 (7.7%)
Ever used modern family planning methods			
Yes	11 (42.3%)	9 (69.2%)	2 (15.4%)
No	15 (57.7%)	4 (30.8%)	11 (84.6%)

Notes: Modern family planning methods (available in the local setting) were defined as: oral pills, intrauterine device (IUD), injectables, implants, and consistent condom use. Non-reversible methods are not included in this list since participants had an unmet need for family planning

Fifteen key informant interviews (KIIs) with community stakeholders who were identified and recruited with help from the ISC were also conducted including: district health officials, family planning providers, VHTs, and cultural, religious, and political leaders from the selected communities. KII participants were compensated 25,000 Ugandan Shillings (~ 7 USD) for their time. All FGD and KII participants provided written informed consent. See Table 2 for an overview of KII participants.

**Table 2:** Key informant interview participants by village, Uganda 2020, N = 15

Community position	Gender
**Village 1**	
HCIII In-Charge/Clinical Officer	Male
HCIII Family Planning Focal Person	Female
VHT Coordinator	Male
Local Council Chairperson	Male
Local Vice Chairperson	Male
**Village 2**	
HCIII In-Charge/Clinical Officer	Male
HCIII Family Planning Focal Person	Female
VHT	Male
Local Council Representative	Female
Muslim Community Leader	Male
**Village 3**	
HCIII In-Charge/Clinical Officer	Male
HCIII Family Planning Focal Person	Female
VHT	Female
Local Council Representative	Female
Muslim Community Leader	Male

Notes: HCIII = Health Centre III; VHT = Village Health Team; local council chairpersons and representatives are elected political officials

Data from these interviews were transcribed, translated, and summarized. Data were analyzed thematically [27]. Through an iterative review of the transcripts by the investigative team (CM, KMS, SMK) we developed a coding guide informed by the social ecological model to classify barriers and facilitators to contraceptive use in order to inform the development of intervention content. Our specific research questions on the development of intervention content and procedures were used to organize data specific to intervention refinement. Two trained research assistants used an iterative process to apply codes manually within the transcripts and coded narratives were extracted and organized in a Microsoft Excel spreadsheet. Coders met weekly with KMS to discuss new codes and potential themes, and to resolve discrepancies through discussion and consensus. The coders independently coded the transcripts deductively following the coding scheme. New codes drawn inductively from the data were created at this stage. KMS reviewed all excerpts after data were fully coded for consensus or re-coding. Codes that represented thematic elements were collated within the spreadsheet and a final round of review/revision of coded excerpts was conducted to confirm thematic validity. To answer the specific questions relevant for intervention refinement, KMS, CM, and SMK independently created initial impression summaries outlining major themes based on review of the coded excerpts. Through several rounds of discussion and revisions between KMS, CM, and SMK, they merged their separate summaries into one finalized summary of thematic results with representative quotations.”

After completing the analysis of the formative research, we convened a hybrid in-person/virtual meeting (due to COVID-19) in October 2020 with the ISC (see Stage 3 in Fig. 2). The investigative team presented a summary of the primary findings of our qualitative data to the ISC members. In this meeting, we gathered the ISC’s input on the interpretation of our qualitative findings and elicited further feedback on outstanding questions specific to intervention content and procedures.

Using the qualitative research findings and ISC feedback, the research team subsequently refined the intervention protocol outline including the proposed activities per intervention session. This outline was further revised based on an additional round of review and feedback from the ISC, as well as review from the broader investigative team. The intervention protocol was finalized, and the associated training manuals developed and later shared with ISC members for final review.

Finally, the manuals were piloted by CM and two trained facilitators with a single group of community members (7 couples) (Stage 4 in Fig. 2). Couples were identified and recruited with help of the community health worker from Wakiso, a neighboring district with characteristics relatively similar to the study district chosen for the larger intervention pilot in December 2020.

Overall, the pilot group sessions had 14 participants (7 couples); the majority were aged 25-34 (n = 8, 57%), Muganda by tribe (n = 9, 43%), Christian (n = 10, 71%), had attained secondary level of education (n = 7, 50%), and had been married between 1 and 5 years (n = 10, 71%), as described in Table 3.

**Table 3:** Participant characteristics for small pilot group sessions, Uganda, 2020, N = 14 (or 7 couples)

Variable	Category	Women	Men	Total n (%)	
Age in years	25–34	5	3	8 (57%)	
	35–49	2	4	6 (43%)	
Tribe	Muganda	5	4	9 (64%)	
	Musoga	0	2	2 (14%)	
	Munyankore	2	1	3 (21%)	
Religion	Christian	5	5	10 (71%)	
	Muslim	2	2	4 (29%)	
Education level	No grade	1	1	2 (14%)	
	Primary	3	2	5 (36%)	
	Secondary	3	4	7 (50%)	
Years married	1 to 5	5	5	10 (71%)	
	6 to 10	2	2	4 (29%)	

Each intervention session during the small pilot was audio-recorded and transcribed for investigators to review and give final feedback to the facilitators on the delivery of the materials (e.g., fidelity to the protocol) and make final adjustments. Through this pilot, we sought to assess the facilitators’ accurate delivery of the session content, their experience with the manuals/study materials (e.g., ease of use, flow of sessions), the response from participants (e.g., active engagement, comprehension, issues within couples), the perceived acceptability of the content to participants, and to identify any other issues with implementation (e.g., the total time of sessions). These issues were assessed through CM’s direct observation of sessions, the investigative team’s review of the session transcripts, and feedback from the facilitators, all of which were considered and discussed by the investigative team. The intervention was finalized and delivered thereafter by two trained intervention facilitators in the planned larger trial (evaluation reported in another paper) [26].

## Results

Overall, the qualitative data supported the proposed intervention approach and informed the development and refinement of intervention content and procedures to increase acceptability and feasibility. Next, we highlight the key findings and adaptions made based on these findings, presented in summary form below and with additional details and select illustrative quotes in Table 4.

**Table 4:** Key Findings from community-engaged research methods to develop the family health = family wealth intervention, 2020, Uganda

Key Findings Used to Develop and Refine the Intervention with Select Representative Quotations	Integration/Adaption into Content
**Data identified specific community norms and beliefs that influence large family size and impede contraceptive use that need to be reshaped through transformative communication to increase family planning acceptance. Key beliefs identified and included in intervention content are listed below:**	
*Beliefs to reshape among both men and women* • Each child brings their own “luck,” therefore, one must have many children to increase the chances of having a lucky (or successful) child • Women’s/Men’s status is tied to the number of children they have • It is a women’s role to take care of children, while it is a man’s role to provide for the family • It is a man’s final decision on whether a couple should use family planning. If he does not want his wife to, she must obey • Contraceptive methods have dangerous side effects and reduce women’s sex drive *Beliefs to reshape among men only* • A man must continue the clan and match the number of children his father had • Men must have children from multiple women to increase the chances of a “lucky” child • Men are meant to have more than one wife, and therefore should not limit their number of children • A woman who is using family planning is probably unfaithful to her husband *Beliefs to reshape among women only* • When your relationship is in trouble, having a child will help save the marriage • Having a child to please your husband will prevent him from having children with other women	• In facilitated community dialogue in Session 1, facilitator presents each belief and guides participants to identify how these beliefs can hurt “family health and wealth” – together the group reshapes the belief to align with gender equity and family planning (women and men’s separate groups) • Specific contraceptive method myths and misinformation identified debunked through family planning education provided by the midwife in session 2 (women only) and session 3 (couples session)
**Strategies to engage men in intervention sessions and increase their acceptance of family planning**	
*Men respect the opinions of community leaders and are influenced by them* • Mobilization of men should involve respected leaders in the community	• Community leader endorsement of the program and family planning integrated at the beginning of the program (Session 1) and the end of the program (Session 4)
*Men are interested in the economic benefits of family planning* • The economic benefit of family planning was the primary facilitator identified for family planning acceptance among men. • Men have a general interest in learning about economic development; greatest interest was expressed in the proposed content focused on “economic health” among men o “*Men are always pre-occupied with wanting to find ways of making money to cater for their families. So, within the topics you are planning, make sure that in the men’s session, you include one which caters for income generating ventures, that seeks to improve the standard of living in families*.” (Community Leader KII)	• The benefits of family planning to “economic health” promoted throughout the program • Economic training (budgeting, advice from a local business expert) included in Session 2 and Session 3 to engage men’s interest
*Men will not attend sessions if packaged as a “family planning” program* • Family planning viewed as a “women’s issue,” making men unlikely to attend a “family planning” intervention o “*So, my husband will come for the first session but will not come back for the second session once he hears about family planning issues. He will think it is for women.*” (Women’s FGD) • Needs to be packaged in a way that makes family planning secondary o “*It is a good program and good to participate in but you have to start with these other components [economic content, etc.] you have mentioned then later you bring in family planning. If you don’t do that, you will not get respondents.*” (Men’s FGD)	• Family Health = Family Wealth theme used throughout, focused on physical, economic, and relationship health, with family planning highlighted as important to all three areas • “Family Planning” redefined as being broader than contraceptive use, but planning for one’s family in all three areas of health
*Men will expect incentives to attend* • Small incentives typically given for attendance of community meetings, and therefore expected • ISC confirmed that community dialogues by the health facility would include a small monetary incentive, deemed scalable within health system if small ~(5,000–10,000 Ugandan Shillings)	• 5,000 Ugandan Shillings provided for attendance of each session
**Acceptability of community leader participation**	
*Community leader participation in the intervention viewed as an effective way to endorse the program and increase family planning acceptability to community members* • Participants agreed that community leader endorsement of the program and family planning would improve community acceptance of the intervention and contraceptive use o “*In our community, the local council chairmen are highly listened to. Their opinions matter to the people. The people are used to them and believe in them*.” (Village Health Worker KII)	• Religious and elected leaders identified to endorse the program is Sessions 1 and 4 • Local leaders with expertise in intervention content selected to co-facilitate specific intervention content following a script o Midwife: Family Planning Education (Session 2, women and Session 3, couples)
• Influential leaders identified that would be willing to endorse program included: Christian and Muslim leaders, local elected leaders, leaders within the health system, and local business people *Leaders can endorse the program, and leaders with specific expertise can co-facilitate content-specific session, but should follow a specific script to stay on message* • Mobilizing and co-facilitating scripted aspects of the session considered an appropriate role, but not leading sessions directly as originally planned • Important to ensure the intervention was not viewed as politically affiliated (with elected leader involvement), making it important to control leader messages through intervention scripts	o Local Business Experts (male and female): Advice on Starting a Family Business (Session 2, men and women’s groups) o Community Development Office: Community Action Plan (Session 4, couples)
**Managing gender dynamics and minimizing risk of unintended negative consequences of participation**	
*Concern was raised about content creating conflict within couple and about women’s ability to openly participate with partner present; strategies to mitigate risk and ensure equitable participation were elicited* • Facilitators will have to meet with men separately first to sensitize them on the content before having couples attend together o “*I see that this kind of strategy [community dialogues] would not be effective unless you first provide counseling and education to men separately and women separately and make sure that their spouses are in agreement*.” (Village Health Worker KII) • Some concern about women’s ability to openly participate in dialogues with their partner present o Content and facilitator training must include efforts to create a safe space for equitable dialogue • For couples where violence is already occurring, concern raised that discussions about family planning and gender equity could increase women’s risk of violence o Need for appropriate training of facilitators to monitor and handle high-risk cases, and for procedures built into study protocol to monitor the occurrence of unintended negative consequences to participation	• Findings confirmed the acceptability of the proposed format, including two gender segregated groups (women and men groups separate) before two gender-integrated groups (groups of couples together), with importance placed on sensitizing men to the content ahead of the gender-mixed groups • Facilitators trained to set tone for equitable participation between couples, and to identify and handle inequitable participation • Intimate partner violence monitoring methods developed to continuously monitor for unintended negative consequences of participation and to identify couples at higher risk based on a history of violence • Data Safety Monitoring Board established to review safety data throughout the trial
*Difficulty engaging couples from polygamous marriages* • Deemed acceptable as long as the woman and man both agree to participation • Barriers to family planning were identified that were specific to a polygamous community, e.g., women’s fear of their spouse finding another wife if she chooses family planning, women deciding to having children to “compete” with co-wives, and men choosing to having children with many women before being able to cater for the ones he has	• Issues related to navigating family planning decision-making within the context of a polygamous community were integrated into intervention content (e.g., promoting being able to care for the children one has before having children with another woman)
**Intervention format and structure**	
*Information elicited to inform the ideal format and structure of the intervention* • Number of sessions: four total sessions acceptable • Gender mixed deemed acceptable (discussed above), as well as mixed ages • Duration of and spacing between sessions: 1 to 1.5 h, 1–2 weeks between sessions • Timing: Most people work in the gardens in the morning; making afternoon ideal • Location: Must be centrally located in the community	• Four sessions (two gender segregated, two gender mixed) conducted 1–2 weeks apart held in the afternoons at a central location like the health facility
**Acceptability and feasibility of linking community-based family planning distribution to intervention sessions**	
*The delivery of short-term contraceptive methods during group sessions is feasible and was deemed acceptable by community members if made explicitly voluntary* • ISC and health workers in KIIs confirmed the feasibility of approach, using only short-term methods (i.e., condoms, oral pill, injectables) • Community members felt approach was acceptable, but should be made optional, at the end of sessions, making it easier to opt out of the service if uninterested.	• Midwife to offer counseling and short-term contraceptive methods after Sessions 3 and 4 (couple sessions) for those who opt to stay after for the service
**The need to strengthen providers’ family planning capacity and monitor family planning stock**	
*Health system gaps that could hinder the effectiveness of the intervention were identified that needed to be integrated into the intervention’s content and study procedures.* • Health workers within the local Health Centre’s did not feel comfortable providing all contraceptive methods and forms of counseling. Specific knowledge gaps identified included intrauterine device (IUD) insertion and removal, as well as how to counsel patients on side effect management. o “*We lack the personnel that is especially skilled in offering those long term methods*.” (Health Worker KII) • Stocks outs of methods were identified as common within the district.	• Intervention content enhanced to address capacity gaps through a 2-day training provided to health care providers at the participating Health Centres to build capacity on the delivery of family planning counseling and contraceptives methods; emphasis on gaps identified, e.g., insertion and removal of IUD • Methods integrated into the intervention trial to monitor the contraceptive stock at the clinics in the intervention and control villages and notify the health district to ensure restock during the intervention trial

### Multilevel approach and need for normative change

The qualitative data and ISC feedback confirmed our hypothesized multilevel barriers to contraceptive use, supporting the overall multilevel approach to target individual, interpersonal, and community-level factors through community dialogues. The findings highlighted specific cultural norms and community beliefs to target for change that influence large family size preference and inequitable decision-making between spouses. Table 4 highlights a selection of key community beliefs identified through the formative work included in the final intervention package to be reshaped to align with family planning acceptance.

### Strategies for male engagement

The qualitative interviews and ISC confirmed the importance and challenge of engaging men in the sessions and of them accepting family planning. Strategies to overcome barriers to male participation were identified: mobilization through community leaders, increasing economic focus of content, packaging of the intervention focus beyond family planning alone, and providing small incentives. These strategies were integrated in the single group, small pilot session with positive results.

### Acceptability of community leader participation

Engaging community leaders in the intervention was deemed acceptable and likely to increase support for the program, as well as family planning; however, we found leaders should serve to endorse the program, but not facilitate dialogues directly as originally proposed. Local content experts (e.g., midwives, local business experts) would be acceptable co-facilitators in sessions specific to their areas of expertise.

### Managing gender dynamics and minimizing risk of unintended negative consequences of participation

With session content focused on family planning and challenging traditional gender norms, a concern was raised that participation in the couple’s sessions could create unintended negative consequences for women, such as conflict with partner or increased risk for intimate partner violence for women already in abusive relationships. Similarly, concern was raised in the qualitative interviews and with the ISC that women might not be able to fully participate with their partner present, as the male partner might dominate the conversation or the woman might fear being honest. Despite these concerns, the overall consensus was that the approach would be acceptable if men were carefully sensitized about the program to start in the first two gender-segregated sessions and if staff were properly trained. The findings also informed the development of methods to be integrated into the standard operating procedures to identify women at heightened risk for violence (i.e., history of violence in the relationship) and to monitor the occurrence of any unintended negative consequences due to participation throughout the study. More details on the risk mitigation strategies developed based on these findings are described in Table 4, which were employed in the single group pilot; no couples reported any increased conflict or violence due to the study in the small pilot.

In addition, the high prevalence of polygamy practiced in the community raised questions about whether recruiting men with more than one spouse into the program would be culturally appropriate and whether it could lead to conflict within couples. However, there was consensus that it would be acceptable as long as both the woman and man were fully informed about the study and agreed to participate. A number of issues affecting large family size specific to families in a community where polygamy is prevalent were raised and integrated into intervention content (see Table 3 for examples).

### Intervention format and structure

The formative work yielded detailed information to guide the implementation of the intervention, such as the ideal group structure (discussed under gender dynamics, four sessions: two gender segregated groups, two gender mixed groups); mix of ages deemed acceptable, timing (afternoons), location (central place in community), and the duration of and timing between sessions (between 1 and 2.5 h, every 1–2 weeks).

### Acceptability and feasibility of linking community-based family planning distribution to intervention sessions

A goal of the intervention is to reduce structural barriers to family planning by creating linkages between the health system and the community dialogues. In the initial development of the intervention protocol, it was unknown whether or not it would be deemed feasible and acceptable to provide family planning counseling services and the distribution of methods directly to participants as part of the sessions. The ISC and KIIs with health workers confirmed that from the District’s perspective, it would be allowable to deliver short-term methods during sessions (i.e., condoms, oral pills, injectables). The focus groups discussions with participants found that this approach would be acceptable to community members, but that it should be made explicit to participants that the service is optional, and it should be delivered at the end of sessions, making it easier to opt out of the service if uninterested.

### The need to strengthen providers’ family planning capacity and monitor family planning stock

Among the primary barriers to contraceptive use that emerged at the health-system level, a gap was identified in health workers’ ability to provide all contraceptive methods, particularly LARCs to patients. Based on these findings, the intervention was modified to include a needs assessment of the public health facilities to assess gaps in contraceptive knowledge and skills among health workers to inform a tailored family planning refresher training provided in partnership with the District Health Team as part of the intervention.

Similarly, issues with contraceptive stock not being always available at the local clinics were shared. This finding highlighted the need for the study to develop methods to monitor stock at the clinics of the participating communities in the pilot trial, and work with the district to fill gaps if identified during the trial.

### Overview of final intervention package

An overview of the final intervention package informed by the data described above is presented in Table 5. The final package includes a total of four sessions, two gender segregated and two gender mixed. All sessions are to be delivered by two trained intervention facilitators and to take place approximately one to two weeks apart from one another. The planned theme of “Family Health = Family Wealth” remains throughout the content, with content developed to enhance all three areas of health (physical, relationship, economic), with family planning integrated into each area as key to achieving family success within that area.

**Table 5:** Overview of finalized content of the *family health = family wealth intervention, organized by the three areas of “Family Health”: physical health, relationship health, and economic health*

Session	Outlined content
**Pre-intervention health worker capacity building**	• Needs assessment conducted at public health facilities in intervention village to assess gaps in contraceptive knowledge and skills among health workers conducted in partnership with District Health Team. • Tailored family planning refresher training provided in partnership with the District Health Team to address training gaps.
**Session 1**	
**Men’s Only Session** *~ 90 min*	• Guided discussion to identify gender-specific definitions of “family wealth,” interpersonal and community barriers to family health and wealth, and redefine group norms on a “successful” family. Content tailored to the norms relevant to men and women’s separate groups. • Program and family planning endorsed by a community leader
**Women’s Only Session** *~ 90 min*	
**Session 2**	
**Men’s Only Session** *~ 2 h*	• Relationship Health: Discussion on healthy relationships and family planning (partner violence, communication, decision-making, caregiver roles, gender norms); role modeling of gender equitable couples • Economic Health: Business skill training co-facilitated with a local business expert (male expert)
**Women’s Only Session** *~ 2 h*	• Physical Health: Contraceptive education co-facilitated with a health worker • Economic Health: Business skill training co-facilitated with a local business expert (female expert)
**Session 3**	
Couples’ Session *~ 2 h*	• Physical Health: Contraceptive education co-facilitated with a local health worker; Health worker to provide family planning/linkages to care; create a “Family Action Plan” – setting family size and contraception goals • Relationship Health: Communication skills building activities; create a Family Action Plan – setting relationship goals (take home assignment) • Economic Health: Family budgeting
**Session 4**	
Couples’ Session *~ 2 h*	• Relationship Health: Communication skills building activity • Revisit Family Action Plan goals as a couple • Guided discussion to identify community barriers and solutions for family planning access/uptake co-facilitated with community leader (e.g., Community Development Officer) • Introduction to a “Community Action Plan” co-facilitated • Local health worker to provide family planning/linkages to care • Program and family planning endorsed by a community leader

Notes: Total of four sessions, two gender segregated and two gender mixed, all sessions to be delivered by two trained intervention facilitators and with co-facilitators from the community (i.e., local health worker, local business expert, community leaders) as specified in the table. All co-facilitators will be trained in the intervention content and will be provided a manual with a suggested script to follow. Sessions are planned to take place approximately 1–2 weeks apart from one another

## Discussion

This manuscript describes the development of the content and procedures of a multilevel, community-based family planning intervention designed for couples in rural Uganda that has been piloted and evaluated with promising results [26]. Informed by the formative work described in this manuscript, the final intervention package is comprised of multiple group sessions (2 gender segregated, 2 gender mixed) aimed to address multilevel barriers to contraceptive use, including community dialogues with groups of couples to reconstruct group norms enhanced with activities to improve knowledge, motivation, couple dynamics, and link couples to services. The original intervention plan was adapted to strengthen its potential effect on health system barriers to contraceptive use through the development of a targeted needs assessment and refresher training of healthcare workers (HCWs) in the intervention community in family planning methods, and through the direct distribution of short-term contraceptive methods during group sessions (resulting in an addition to the original conceptual model displayed in Fig. 1). The HCW training content developed includes general education on contraceptive methods and practical skills in how to counsel and provide the methods to clients, with an emphasis on filling identified gaps in the provision of LARCs.

While the intervention’s preliminary effectiveness is yet to be determined, the findings of this study may still have implications for the development of multilevel interventions aimed to increase contraceptive use in settings similar to this rural community in Uganda. The community dialogue approach that is part of the proposed intervention has been widely used by multinational agencies for reproductive health programming, [19] but has not been rigorously tested and published in peer-reviewed literature [25]. Successful examples demonstrate improvements in equitable relationships, community gender norms, and community ownership of a problem, but mainly focus on HIV and rely on qualitative methods [20, 28–35]. One intervention in Kenya provides stronger evidence for gender-focused community dialogues: participation was associated with 1.78 times higher odds of contraceptive use post-intervention for women, but notably, was not effective for men [36]. Our approach to enhance the effect of community dialogues by linking them with other multilevel approaches may be needed to engage men and address relationship and community drivers of family planning. Our community-engaged methods identified specific community beliefs/norms to be reshaped by our dialogue, many of which center on gender inequitable norms. Evidence from randomized controlled trials in sub-Saharan Africa support similar “gender transformative” communication in HIV risk and intimate partner violence reduction [37–39].

Consistent with the findings of our study, male partner disapproval of family planning is a common barrier to contraceptive use in LMICs [40, 41]. While increasing men’s acceptance of family planning and engaging men in family planning interventions can be a challenge, [42, 43] men often express a strong interest in learning more about family planning and want to be involved in reproductive decision-making [44, 45]. This formative qualitative work presented here offers a number of strategies to increase male engagement, such as framing the intervention around men’s interests, mobilizing men through community leaders, and providing small incentives for participation. It also generated strategies that will be tested in the full pilot to ensure women’s safety and full participation with their partner present. Similar strategies to engage men have gained support through other research, such as engaging men’s interest by promoting the financial benefits of family planning and having male champions for family planning encourage men’s participation [45, 46]. However, reviews of male engagement strategies conclude that evidence is still accumulating and strategies need to be tailored the cultural context of each community, [47–50] making the findings of the present study an important addition to the literature.

This study also provides preliminary support for the pairing of community dialogues that increase family planning demand with community-based family planning (CBFP) delivery methods. The formative work presented here found the delivery of short-term methods during the planned group sessions feasible from the health system’s perspective, and potentially acceptable to community members. CBFP methods are an effective strategy to scaling up contraceptives in rural areas where structural barriers like geographic distance and long wait lines impede uptake, and Uganda has pledged to scale up CBFP as part of their FP2030 strategy [51]. Moreover, this approach may be important to explore in the context of COVID-19 outbreaks and related lockdowns preventing communities from receiving family planning from facilities [52]. However, CBFP efforts need to be paired with demand generation activities to optimize their effect, while also addressing the structural barriers identified in our study related to stock out and low health worker capacity to provide LARCs.

Our study’s findings may not be generalizable to dissimilar settings. However, the multilevel barriers that our intervention aims to address are common across settings in sub-Saharan Africa and East Asia, making our findings potentially applicable to settings where the high unmet need for family planning is similarly tied to gender norms, relationship equity, and community dynamics and where community-based health service models are utilized.

While this study is limited in its relatively small sample, saturation was reached, and the findings aligned with and expanded on our preliminary research with this population [16–18]. Limitations to the intervention approach itself are detailed with the pilot evaluation [26]. Despite support for our overall approach, couples-based family planning interventions such as ours need to prioritize the mitigation and monitoring of unintended consequences related to partner violence and unintended reproductive coercion. Our study’s strength is its use of a series of iterative approaches that involved feedback at multiple points from a range of community stakeholders; the methods used can serve as a model for other studies aiming to develop and refine an intervention for a specific setting. Community-engaged research is recognized as key to gaining community participation and trust, developing acceptable, feasible and effective programs, and translating research into real-world health programs [53–55]. In the subsequent pilot of the Family Health = Family Wealth intervention, the ISC was engaged throughout the study [26], and a process evaluation was conducted to further understand barriers to implementation and future adoption (to be published separately), so that the content can continue to be improved to fit the local context.

## Conclusion

The Family Health = Family Wealth intervention is a community-based, multilevel family planning intervention that engages groups of couples in transformative dialogues, while addressing key individual-, interpersonal-, and health-system-barriers to family planning. The feedback elicited from community participants largely supported the planned intervention content and structure, but the data provided additional direction for further development of the intervention content and procedures. Key findings that informed intervention development included the inclusion of locally derived community beliefs to reshape through transformative communication, strategies to engage men, acceptable approaches to community leader involvement, strategies to manage gender dynamics and ensure participant safety, the delivery of contraceptive methods directly to participants during community dialogues, and the inclusion of intervention components to strengthen providers’ family planning capacity and monitor family planning stock. This study’s findings may be informative for the development of family planning interventions in similar settings, and the methods described may also serve as a model for other researchers in the application of community-engaged methods to develop or refine and adapt an intervention for a specific community. The resulting intervention package is currently being pilot tested for acceptability, feasibility, and preliminary effects on contraceptive use and related outcomes among couples with an unmet need for family planning.

## Data Availability

For ethical reasons, the data used to support this study are not available. The raw, qualitative data collected from key stakeholders interviewed would be difficult to fully de-identify given that the study location is relatively small and has been publicized elsewhere. To protect confidentiality, the authors opt not to share the dataset. However, cmuhumuza@musph.ac.ug the corresponding author is the point of contact regarding access and issues related to data.

## Declarations

### Paper context

Family planning has important implications for public health, social, and economic outcomes, but few interventions exist to address multilevel barriers to contraceptive use in low-income settings. This study reports on the development of a multilevel community-based intervention through community-engaged methods to inform intervention content and procedures. The resulting intervention package that will be pilot tested is presented and the findings could inform intervention development for similar settings.
